# Nutritional, Fatty Acids, (Poly)phenols and Technological Properties of Flower Powders from *Fuchsia hybrida* and *Alcea rosea*

**DOI:** 10.3390/foods13020237

**Published:** 2024-01-11

**Authors:** Maritza Castillo-Carrión, Ruth Martínez-Espinosa, José Ángel Pérez-Álvarez, Juana Fernández-López, Manuel Viuda-Martos, Raquel Lucas-González

**Affiliations:** 1 Departamento de Química, Facultad de Ciencias Exactas y Naturales, Universidad Técnica Particular de Loja, Loja 110108, Ecuador; mjcastillo1x@utpl.edu.ec (M.C.-C.); rimartinez@utpl.edu.ec (R.M.-E.); 2 IPOA Research Group, Centro de Investigación e Innovación Agroalimentaria y Agroambiental (CIAGRO-UHM), Miguel Hernández University, 03312 Alicante, Spain; ja.perez@umh.es (J.Á.P.-Á.); j.fernandez@umh.es (J.F.-L.); mviuda@umh.es (M.V.-M.); 3 Centro Tecnológico de la Carne de Galicia, Parque Tecnológico de Galicia, Avda. Galicia No. 4, 32900 Ourense, Spain

**Keywords:** flower podwer, pena pena, malvagoma, horchata drink, anthocyanin

## Abstract

*Fuchsia hybrida* (pena pena) and *Alcea rosea* L. (malvagoma) are predominant flowers in the “Horchata” infusion, a traditional beverage in southern Ecuador, to which some medicinal properties are attributed. However, there is very little published information about these two flower species. The current study aimed to obtain two dehydrated powders of these flowers and to determine their chemical composition, physicochemical and technological properties, polyphenols, and fatty acids profile. In both powdered flowers, carbohydrates predominated, with a significant content of dietary fiber and fructose. The fat content was low, mainly comprising polyunsaturated fats (62% pena pena and 52% malvagoma), with a significant presence of omega-3 (C18:3n-3,6,9) and omega-6 (C18:2n-6,9) fatty acids, showing a better n-6/n-3 balance in the malvagoma flowers. Pena pena flowers are highlighted by high anthocyanin and ellagic acid amounts, whereas malvagoma contains a high content of flavanones. In conclusion, the studied powder flowers, could be used in the formulation of new foods or as source of anthocyanins as food colorants.

## 1. Introduction

Horchata is a popular herbal infusion made from a mixture of 16 to 32 medicinal plants, commonly consumed in Southern Ecuador for its anti-inflammatory, analgesic, and diuretic properties [1,2]. Among the 20 culturally significant medicinal plant species used in this beverage, *Alcea rosea* and *Fuchsia hybrida* play a predominant role. *Fuchsia hybrida* Hort. T. ex Siebert & Voss, commonly known as “pena pena grande” or “pena pena roja”, is an introduced shrub that imparts aroma to the horchata drink and has various therapeutic uses, including anti-inflammatory, antiflu, cardiotonic, sedative, and for stomachaches [1]. On the other hand, *Alcea rosea* L. (also known as “malva goma”, “malva rosa”, or “malvón”) is an introduced herb that adds flavor to the drink and is known for its therapeutic properties such as expectorant, cooling, analgesic, anti-inflammatory, depurative, diuretic, and tonic effects [1,3].

Flowers constitute a vital component of plants, containing a wide array of bioactive compounds, including phytochemicals such as flavonoids (especially anthocyanins), phenolic acids, carotenoids (carotenes, xantophylls), chlorophylls, and numerous other constituents [4,5,6,7]. Extensive research has been dedicated to examining these components, driven by their profound impact on human health and their possession of antioxidant, anti-inflammatory, anticancer, anti-diabetic, and cardio-protective properties [8,9]. Edible flowers of various varieties have been studied worldwide to elucidate their nutritional composition, encompassing antioxidants, ascorbic acid, anthocyanins, phenolic compounds, carotenoids, chlorophylls, flavonoids, fatty acids, free sugars, vitamins, carotenoids, minerals, and organic acids [4,6,9,10,11,12]. Generally, the content of common components such as lipids, proteins, carbohydrates, and vitamins is like that found in vegetables [13].

Changing consumer habits and lifestyles have created a demand for functional and healthy foods, and natural additives [7,14], as consumers seek healthier and more attractive food options to improve their dietary aesthetics and diversify their sources of micronutrients [15]. In this context, edible flowers have emerged as a new trend in human nutrition. A recent review describes the use of edible flowers in the development of functional dairy products, either to fortify them or as a substitute for natural colorants in products such as yogurt, milk, curd, and probiotic drinks, incorporating flowers in the form of extracts, distilled water (maceration), powder, or syrup [16]. Gamage et al. [17] studied the blue pea flower for its significant content of delphinidin-3-(trans-p-coumaroyl)-glucoside, the predominant anthocyanin in this flower, and obtained a natural colorant that is more stable under thermal and refrigeration treatments than spirulina, which is the only approved natural food colorant in Europe. On the other hand, Hnin et al. [18] used rose flower powder as an ingredient to develop nutritional cookies at different levels. They demonstrated that the flower powder offered positive nutritional and sensory properties, including higher total phenolic and total anthocyanin contents, increased antioxidant activities, a higher color score, and an improved odor of cookies due to the associated rose flower fragrance.

In many countries worldwide, they are consumed for their potential health benefits or as part of traditional foods to enhance nutritional value, add fresh and exotic aromas, delicate flavors, visual appeal, and vibrant colors [19,20]. Furthermore, the utilization of these species can contribute to the valorization of local flora through the large-scale production of edible flowers, considering the increasing global demand for edible flowers and the growing number of producers and points of sale, including supermarkets, local markets, and online outlets [8,21]. The edible flowers segment holds value in the business market, as the industrial sector explores various possibilities for incorporating bioactive compounds from edible flowers into new food products. This can be achieved through the direct use of various floral parts (petals, stems, sepals) or by incorporating their extracts or essential oils [8,22]. The search for new food products also involves seeking new colors, textures, and flavors that can be achieved using edible flowers [23]. The edible flowers sector for human consumption is diverse, encompassing a wide range of value-added alternative food products, including drinks, beverages, dishes, preserves, jams, and more [24]. The food industry stands to benefit by meeting market demands for functional and healthy foods through the development of novel floral-based foods and formulations, thereby allowing for the valorization of previously unexplored or underexplored species [21]. Therefore, the current study aimed to obtain two flower powders from pena pena (*Fuchsia hybrida*) and malvagoma (*Alcea rosea*) and to determine their chemical composition, physicochemical and technological properties, and (poly)phenol and fatty acid profiles. The study results allowed the functional potential of both flowers to be revealed for use in the development of new foods.

## 2. Materials and Methods

### 2.1. Plant Material

The pena pena (*Fuchsia hybrida*) and malvagoma (*Alcea rosea*) flowers (Supplementary Figure S1) were purchased from a local market in Loja, Ecuador. The freshest flowers that were not wilted were selected and immediately dehydrated (40 °C, 36 h) in a tray dryer, Model DY-110H (LASSELE.CO, Danwon-gu, Republic of Korea). Subsequently, they were ground in an ultracentrifugal mill ZM 200 (Retsch GmbH, Hann, Germany) until obtaining a final particle size of <210 µm. The powdered flowers were vacuum sealed and packaged in hermetically sealed aluminum bags to protect them from light and moisture.

### 2.2. Chemical Composition

AOAC methods [25] were used to determine the moisture (No. 925.09), ash (No. 925.09), fat (No. 948.22), and protein (No. 935.11) content in the samples. Results were expressed as g/100 g of dehydrated powder. Dietary fiber (TDF) was determined using an enzymatic-gravimetric method based on the AOAC official method No. 985.29. Total carbohydrates were calculated by the difference.

### 2.3. Sugar Content

The sugar profile of powder flowers was carried out through HPLC following the method described by Lucas-González et al. [26]. In brief, the samples were homogenized with ultrapure water in an Ultra-Turrax at 12,000 rpm for 60 s with a solid–solvent relation of 1:40. Next, the samples were centrifugated at 5000× *g* for 10 min at 4 °C. Then, the supernatant was collected and filtered through a 0.45 μm Millipore filter (Millipore Corporation, Bedford, MA, USA). Detection and identification of samples were carried out through HPLC (Hewlett-Packard HP-1100 instrument (Hewlett-Packar, Woldbronn, Germany)) coupled with a UV–visible diode array detector G1315A (set at 210 nm) and a refractive index detector G-1362. A cation exchange column (Supelcogel C-610H, 300 × 7.8 mm; Supelco, Bellefonte, PA, USA) with a pre-column (Supelguard-H, 50 × 4.6 mm, Supelco) was used to separate compounds using a mobile-phase phosphoric acid (0.1% *v*/*v*). The HPLC conditions were: injected volume, 10 µL; flow rate, 0.5 mL min^−1^; temperature, 30 °C; and run time, 30 min. Standard glucose, fructose, and sucrose were used to identify compounds by comparing their retention time with sample peaks. A regression formula of standards was used to quantify sugar in samples. The sugar content was expressed as g 100 g^−1^ of sample.

### 2.4. Fatty Acid Profile

Fat extraction was carried out as follows: 20 g of samples was mixed with hexane (1:4; *w*/*v*). The solution was submitted to one hour of ultrasonic bath plus 30 min of magnetic agitation. Then, the samples were centrifugated (10 min; 4 °C; 7200× *g*). The supernatant was evaporated under vacuum and the fat was resuspended in a 2–3 mL of hexane and filtered through a 0.45 μm Millipore filter (Millipore Corporation, Bedford, MA, USA). The hexane was evaporated again under vacuum and the remaining fat (around 100 mg) underwent a methylation procedure, previously described by Lucas-González et al. [27]. A Gas-Chromatographer HP-6890 (Woldbronn, Germany) equipped with a flame ionization detector (FID) and a Suprawax 280 capillary column (30 m × 0.25 μm film thickness × 0.25 mm i.d.; Tecknokroma, Barcelona, Spain) was used to identify fatty acids in the samples using the same conditions proposed by Botella-Martínez et al. [28]. Fatty acid methyl esters (FAMEs) standards (Supelco 37 component FAME Mix, Bellefonte, PA, USA) were used to identify compounds by comparing their retention time with the sample retention time. The results were expressed as mg/100 g of sample.

### 2.5. Free and Bound (Poly)phenol Profile

For the extraction of free (poly)phenols, the methodology described by Lucas-González et al. [27] was followed, while for obtained bound (poly)phenols, the procedure proposed by Mpofu et al. [29] with the modification carried out by Lucas-González et al. [27] was used. In brief, two grams of the samples were submitted to two extraction steps in sequence, first with an aqueous–methanol (20:80 *v*/*v*) and then with aqueous–acetone (30:70 *v*/*v*). In each extraction process, samples were sonicated (10 min in ultrasonic bath), centrifugated, and the collected supernatant evaporated under vacuum. The dry extract was resuspended in water and then purified with a C-18 Sep-Pak cartridge. The free (poly)phenols were collected in acidified methanol (MeOH: formic acid; 99:1; *v*:*v*). The pellet was subjected to an alkaline–acid extraction to extract bound (poly)phenols. In brief, the pellet was mixed with 50 mL of 4 M NaOH and left in darkness for 4 h. Afterward, the medium was acidified (pH 2.0) with 6 M HCl. The sample was centrifuged, and the supernatant was extracted with ethyl acetate. The organic solvent was dried under vacuum, and the sample was resuspended in methanol and filtered thought a 0.45 μm Millipore filter (Millipore Corporation, Bedford, MA, USA).

A Hewlett-Packard HPLC series 1200 instrument (Woldbronn, Germany) equipped with UV–visible diode array detector and coupled with a C18 Teknokroma column (Mediterranean sea18, 25 × 0.4 cm, 5 μm particle size; Teknokroma, Barcelona, Spain) was used to detect and elute compounds. The mobile phases were formic acid in water (1:90, *v*/*v*) as solvent A and acetonitrile as solvent B. The working conditions were 180 bar, gradient elution at 1 mL min^−1^ with the following gradient program: started with 95% A, 75% A at 20 min, 50% A at 40 min, 95% A at 45 min and 20 µL of injected volume. Four wavelengths were used, 280, 320, 360, and 520 nm, for the detection of (poly)phenols. The identification of compounds was performed by comparison of their retention time and absorbance spectrum with the standards (gallic acid, 4-hydroxycinnamic acid, ellagic acid, protocatechuic acid, sinapic acid, syringic acid, vanillic acid, vanillin, ferulic acid, caffeic acid, p-coumaric acid, chlorogenic acid, rosmarinic acid, catechin, epicatechin, gallocatechin gallate, gallocatechin-3-gallate, catechin-3-gallate, epicatechin-3-gallate, epigallocatechin-3-gallate, quercetin, kaempferol, myricetin, rutin, apigenin, luteolin, luteolin-7-O-glucoside, naringenin, hesperidin, neorecitrin, and neohesperidin, cyanidin-3-O-β-glucopyranoside, delphidin-3-O-β-glucopyranoside, malvidin, malvidin-3,5-O-β-glucopyranoside, malvidin-3-O-β-glucopyranoside, pelargonidin-3-O-β-glucopyranoside, peonidin-3-O-β-glucopyranoside, petunidin-3-O-β-glucopyranoside), which were eluted in the same conditions of samples. Quantification was carried out using a standard regression formula. The results were expressed as µg/g of sample.

### 2.6. Physicochemical Properties

A 10% (*w*/*v*) aqueous solution of flower powder samples was used to determine pH with a pH meter (pH/Ion Model, Eutech Instruments Pte Ltd., Singapore). Water activity was measured in Sprint TH-500 Novasina Thermoconstanter at 25 °C (Lachen, Switzerland). The color coordinates, L* (lightness), a* (±red-green), and b* (±yellow-blue), were measured in the CIELab color space with the help of CM-2600d colorimeter (Minolta Camera Co., Osaka, Japan). The colorimeter was used in SCI mode, D65 as an illuminant, and 10° as an observer. The aperture for illumination and measurement was 11 mm and 8 mm, respectively. The colorimetric values hue (h* = tan^−1^ b*/a*) and chroma (C* = (a*2 + b*2)1/2) were also calculated. Bulk density was expressed as weight of the sample in kg per unit volume of the powder flower (kg/m^3^).

### 2.7. Techno-Functional Properties

Oil holding capacity (OHC) was determined by mixing the sample with vegetable oil (1:10, *w*/*v*) for 30 min. After centrifugation at 4750 rpm using a CLAY ADAMS^®^ Brand DYNAC^®^ (Becton, Dickinson, MD, USA) centrifuge, the OHC was expressed as grams of oil held per gram of the sample [30]. Swelling capacity (SWC) was measured following the method described by Robertson et al. [31] with slight modifications. In brief, 0.1 g of the sample was weighed in a 10 mL graduated cylinder (graduated to 0.1 mL), and 10 mL of distilled water was added. The mixture was gently stirred and left at room temperature (20 °C) for 16 h. Then, the volume (mL) occupied by the sample was measured, and SWC was expressed as mL/g of sample.

### 2.8. Statistics Assay

The results are presented as mean ± standard deviation (SD). Each determination was carried out in triplicate. A one-way ANOVA was carried out using the statistical software SPSS 19.0 (SPSS Inc., Chicago, IL, USA) to compare results between samples. Tukey’s post hoc test was applied for comparisons of means. Significant differences were considered *p* < 0.05.

## 3. Results and Discussion

### 3.1. Chemical Composition

The chemical composition of both powder flower samples is presented in Table 1. Malvagoma flower powder exhibited the highest content in fat, proteins, ash, and fiber (*p* < 0.05). In contrast, the pena pena flower powder showed the largest amount of total carbohydrates and the monosaccharides fructose and glucose (*p* < 0.05).

Carbohydrates were the most abundant macronutrients in both dried petals. Considering that the contents of total soluble sugars significantly influence the taste of edible flowers, it was crucial to assess their content [32]. Rivas-García et al. [33] mentioned that the most prevalent monosaccharides in flowers are fructose, glucose, and sucrose. These findings are consistent with our results, where fructose predominates in both species, with the content being significantly higher in the pena pena powder.

The second most prevalent component was protein, followed by ash. The protein content of both studied flower powders, was similar to that reported for flowers such as Arbutus xalapensis (11.3 g/100 g dw) [34], Agave salmiana, Aloe vera, and Myrtillocactus geometrizans with 11.58, 11.85, and 12.53 g 100 g^−1^ dw, respectively [35]. There are also species with much higher protein values, such as Borage with 22.69 g 100 g^−1^ dw [12]. There are very few studies reported in the literature focusing on the amino acid profile of edible flowers and the presence or absence of essential amino acids in the edible flower [7]. Phenylalanine, leucine, and valine are the most abundant amino acids in some edible flowers. However, flowers like Cucurbita pepo, Erythrina americana, Erythrina caribaea, Yucca filifera, and Agave salmiana, lack the amino acid lysine. On the other hand, flowers such as Aloe vera, Euphorbia radians, and Arbutus xalapensis have tryptophan as the limiting amino acid [34].

The ash content of edible flowers is appreciable, and according to Fernandes et al. [36], it exhibits the highest variability in the total content, ranging from 2.6 to 15.9 g 100 g^−1^ dry weight for Madhuca indica and Cucurbita pepo, respectively, among 32 flowers studied. The ash content of the studied flower powders (Table 1) was at intermediate values of those mentioned. Considering that the ash content is related to mineral content, edible flowers have demonstrated significant mineral content, including potassium, calcium, magnesium, sodium, and phosphorus [36,37]. Therefore, further study of these minerals in edible flowers is important, as the mineral content in edible flowers can be higher than that found in common fruits and vegetables typically consumed daily [7].

Fat was the least abundant macronutrient. Although flowers generally have low fat content, other edible flowers with higher fat values have been reported, such as Aloe vera with 4.61 g 100 g^−1^ dw [38], Calendula with 5.33 g 100 g^−1^ dw [23], Borage, and Pansies with 4.93 and 5.17 g 100 g^−1^ dw, respectively [12].

The total dietary fiber content is notable in the two edible flowers, which is higher than that reported for Aloe vera (13.83 g 100 g^−1^ dw) [38] and Viola × wittrockiana Gams (17.24 g 100 g^−1^ dw) and lower than that of *Camellia japónica* L. (54.55 g 100 g^−1^ dw) and *Centaurea cynaus* L. (67.38 g 100 g^−1^ dw) [12]. Jakubczyk et al. [39] reported the fiber content of 12 edible flowers in their study, with *Calendurla officinalis* L. and *Centaurea cyanus* L. standing out at 62.33 and 53.06 g 100 g^−1^, respectively. In all the flowers, the insoluble fraction predominates (ranging from 8.69 to 57.54 g 100 g^−1^). Al-Snafi [3] mentions that Alcea rosea contained mucilages composed of glucuronic acid, galacturonic acid, rhamnose, and galactose, and that they are part of the high-molecular-weight fiber [40]. High fiber content has been associated with beneficial effects on human health, as it minimizes the risks of several conditions. The consumption of soluble dietary fiber (SDF) is a safe way to lower the risk of conditions such as high blood pressure, hypertension, cardiovascular disease, and diabetes [39,41,42].

### 3.2. Physicochemical Properties

The physicochemical properties of both studied flowers are presented in Table 2. The water content in fresh flowers is the main constituent, varying between 70% and 95%, resulting in higher water activity, which makes the edible flowers more perishable, lasting from 2 to 5 days after harvest [7,36,37], as they begin to discolor, wilt, and exhibit tissue darkening [36]. The drying process applied to the samples in the present study allowed for a reduction in water activity, which, together with pH, helps reduce the risk of microbial growth to extend their preservation [43].

The pH values of both flowers are like those reported for *Agave salmiana*, *Aloe vera*, *Erythrina americana*, and *Myrtillocactus geometrizans*, which range between 4.35 and 5.58 [35]. Among the organic acids that determine the pH of flowers are malic, acetic, quinic, citric, and succinic acids, as demonstrated by Fernandes et al. [12] in their study. They found that malic acid was the major organic acid in *Borago officinalis* L., *Camellia japonica* L., and *Viola × wittrockiana* Gams, except in *Centaurea cyanus* L., where succinic acid was predominant. Similarly, Krzymińska et al. [44] found that malonic, succinic, acetic, and citric acids were the major organic acid components in five studied tulip petals.

The density of malvagoma flower powder was the highest, demonstrating that it is denser than pena pena flower powder. Ahmed et al. [45] reported values ranging from 510 to 640 kg/m^3^ and from 440 to 490 kg/m^3^ for Turkish and Indian lentil flours, indicating that with a larger particle size of 210 µm, which was used in our study, higher density values are obtained compared to smaller sizes (105 µm). This could be attributed to the fact that when smaller particle sizes are used, the content of protein and total starch decreases [46]. Knowing the density of a powdered ingredient, along with its moisture content, particle size, and shape, is important because they collectively influence the flowability. Flowability is a parameter that describes the ability to handle the powder for storage, transportation, dosing, and subsequent industrial processes involving its application [47].

Regarding to color attribute, according to Lucas-González et al. [48], the smaller the particle size of the material, the higher the values of lightness and hue, which they achieved with the particle size < 210 µm used in the present study. The L* value of the two flower powders was similar, but the a* value of pena pena flower powder was higher than that of malvagoma flower powder, which is related to the intense red color observed in this flower when fresh, a color that is retained in the dehydrated powder of this flower. The C* value of ‘pena pena,’ which displayed a vibrant color, was higher than that of malvagoma. Anthocyanins, chalcones, aurones, and some flavonols act as major flower pigments in many flowers [49]. Anthocyanins contribute significantly to the red, blue, and purple color of flowers [50].

### 3.3. Techno-Functional Properties

Techno-functional properties are important because they allow for the evaluation of how a particular ingredient behaves in the food matrix or in the body. These properties are influenced by chemical composition, especially the fiber content and particle size [48,51].

The oil holding capacity (OHC) is a property directly related to the cellulose content of dietary fiber [52]. Evaluating this property in an ingredient is important because it provides information about the mouthfeel and flavor retention of foods [42,45]. The values of OHC were similar in both studied flower powders, approaching values reported for other foods such as palm flower and leaves of smooth amaranth with 1.6 and 1.2 g/g, respectively [53]; apple pomace (1.33 g/g) and brewer’s spent grain (1.21 g/g) [54]; byproduct flours of two persimmon varieties (2.15 and 2.26 g/g for ‘Rojo Brillante’ flour and ‘Triumph’ flour, respectively) with a particle size < 210 µm. Our results suggest that the flower powders studied can be used as ingredients to stabilize foods with high fat contents.

Swelling capacity (SWC) is associated with the content of pectin, cellulose, and hemicellulose in the fiber [52]. Therefore, high levels of insoluble fiber, lower bulk density, smaller particle size, and a larger surface area may contribute to a higher SWC [55,56], as observed in the case of malvagoma flower powder, which had significantly higher SWC compared to pena pena flower powder. These values are notably higher than those reported by Requena et al. [53] for palm flower (12.8 mL/g) and smooth amaranth leaves (3.8 mL/g), as well as for commercial cereal fibers such as oat 600 (7.6 mL/g) and wheat (7.06 mL/g) [51]. In contrast, the SWC value for pena pena was like those this author reported for oat 401 (4.98 mL/g) and bamboo (5.69 mL/g).

Sources of soluble fiber are known for their superior hydration properties, including beta-glucan, pectin, gums, mucilages, and inulin. All these fiber components generally can hydrate well in the presence of water and form highly viscous solutions, even forming gels, as in the case of pectins [57,58]. In the case of malvagoma flower powder, which exhibits higher values in soluble water content, this could be related to its content of mucilages [3].

### 3.4. Fatty Acid Profile

The fatty acid profile of both pena pena and malvagoma flower powders can be seen in Table 3. Twenty-five fatty acids were identified and quantified, and malvagoma was the flower with the highest number of fatty acids detected. Both flower powder were rich in polyunsaturated fatty acids, 62% in the case of pena pena and 52% for malvagoma, results similar to those reported by Pires et al. [23] for rose, *Calendula officinalis* L., and *Centaurea cyanus* L. This differs from other flowers such as pansy (*Viola × wittrockiana*) petals [59], *Anchusa azurea*, *Capparis spinosa*, *Cichorium intybus*, *Hedysarum coronarium*, *Malva sylvestris*, *Robinia pseudoacacia*, *Rosmarinus officinalis,* and *Sambucus nigra* [19], where saturated fatty acids were predominant. Monounsaturated fatty acids were the least prevalent type of fatty acids present in both flower powders.

Regarding pena pena, linoleic acid (C18:2n6), followed by linolenic acid (C18:3n3) and palmitic acid (C16:0), were, in decreasing order, the most prevalent. Meanwhile, in malvagoma, the main fatty acids were linoleic acid, palmitic acid, and linoleic acid. Malvagoma oil also presented significant amounts of oleic acid and eicosadienoic acid. In other flowers, the content of palmitic acid was higher than that observed in the studied flowers, such as in the case of *Viola × wittrockiana* petals with a relative percentage of the mentioned acid of 36.41% [59]. In *Helichrysum italicum* flowers, the omega-6 PUFA linoleic acid was the most abundant fatty acid found (22.55% of total FA), and palmitic acid (C16:0) was the major SFA detected with a value of 1.19 μg/mg dw (16.45% of total FA) [60].

The Polyunsaturated Fatty Acid/Saturated Fatty Acid (PUFA/SFA) ratio is an index used to evaluate the impact of diet on cardiovascular health. Therefore, the higher this ratio, the more positive the effect [61]. The value of this index for pena pena was 2.18 and for malvagoma, it was 1.50, values higher than those reported by Fernandes, et al. [12] for blue borage (*Borago officinalis* L.), camellia (*Camellia japonica* L.), blue centaurea (*Centaurea cyanus* L.) and pansies (*Viola × wittrockiana* Gams.), which were below 0.45. Meanwhile, for other food matrices, values have been reported between 0.11 and 2.04 for meat, 0.50 to 1.62 for fish, 0.20 to 2.10 for shellfish, and between 0.02 and 0.175 for dietary products [61].

Another important index calculated is the ratio n-6/n-3 PUFA, which should be considerably improved regarding its potential role in cardiovascular disease. Numerous studies have highlighted the importance of the n-6/n-3 FA ratio, rather than the amount of each single fatty acid individually. Protective effects appear when the ratio is close to unity, while ensuring an adequate intake of essential fatty acids [62,63]. In the present study, the n-6/n-3 ratio for pena pena is 2.10, and for malvagoma, it is 0.71, demonstrating a better balance between these two groups of polyunsaturated fatty acids.

### 3.5. Bound and Free (Poly)phenol Profile of Powder Flowers

Supplementary Table S1 shows the 63 compounds detected in both studied flower powders along with their tentative identification. Among them, 20 were confirmed through a comparison with available standards. The other (poly)phenols were tentatively identified and quantified by comparing their absorbance spectrum with the standard and supporting this with the literature [64].

**Figure 1:**
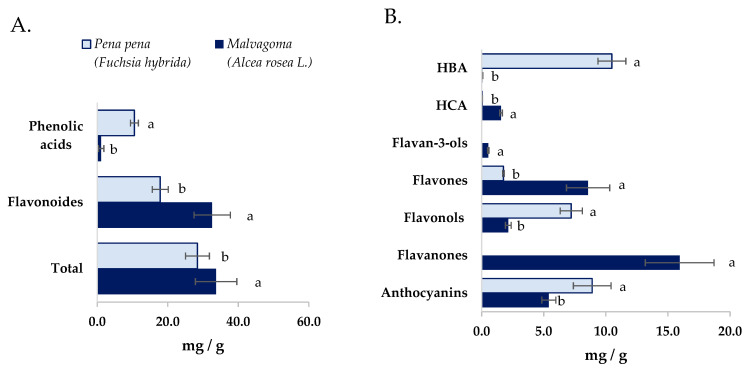
(**A**). Phenolic acid, flavonoids, and total (sum) of (poly)phenols present in both studied flower powders. (**B**). Content of (poly)phenols divided by (poly)phenols sub-families present in both studied flower powders. Values with the same letter in the different figures are not statistically different (*p* > 0.05) for the same tested chemical compound. HBA: Hydroxybenzoic acids; HCA: Hydroxycinnamic acids.

The total amount of polyphenols in both powder flowers samples was similar (*p* > 0.05) (Figure 1A), whereas the polyphenols profile was different between the studied flower powders. Malvagoma flower powder contained the highest diversity of polyphenols sub-families, seven against the four observed in pena pena flower powder (Figure 1B). Nevertheless, both flower powders presented more free polyphenols than bound polyphenols, and a higher number of phenolic acids in bound form than in free (Table 4 and Table 5). That fact could be expected since phenolic compounds are most frequently found in bound forms. Furthermore, in both samples, the hydroxycinnamic acids: ferulic caffeic, and *p*-coumaric, were detected in bound forms. These compounds are frequently found bound to cell walls and proteins in other vegetables like fruits and cereals [65,66].

Concerning the polyphenol profile of pena pena flower powders (Table 4), a total of thirty-five compounds were identified, encompassing ten flavonols, nine hydroxybenzoic acids, six anthocyanins, six flavones, and four hydroxycinnamic acids. Among the free compounds, pelargonidin-3-O-β-glucopyranoside, quercetin glycoside III, ellagic acid, and rutin emerged as the four most abundant, arranged in descending order. In the bound fraction, gallic acid was unequivocally prominent as the most abundant polyphenol. The most remarkable result was the high levels of anthocyanins and free ellagic acid and derivatives. In flowers of *Sanguisorba officinalis,* ellagic acid derivatives as ellagic acid hexosides and 3,3′,4′-*O*-trimethyl ellagic acid [67] were also reported.

Ellagic acid and ellagitannins are abundant in pomegranate, and their metabolic transformation by the human microbiota has been reported as beneficial to health. The content of ellagic acid in pena pena flower powders was close to ellagic acid vegetable sources like raspberry, cloudberry, and strawberry [68] and flowers like *Tagetes erecta* L.

In malvagoma flower powder, twenty-six polyphenols have been observed (Table 5). These include nine flavonols, nine hydroxycinnamic acids, eight anthocyanins, five hydroxybenzoic acids, three flavones, two flavanones, and one flavanol. Notably, in the free fraction, the predominant compounds were naringin glycoside I and II, with luteolin glycoside II following closely. Luteolin glycoside II were also the main compound quantified in bound form, followed by Gallo catechin gallate and caffeic acid. The number of phenolic acids was significantly lower than that shown in pena pena flower powder (*p* < 0.05) (Figure 1A) and observed more diversity of hydrocinnamic acids than hydroxybenzoic acids.

To the best of our knowledge, no previous studies have quantified the polyphenol composition of *Fuchsia hybrida* and *Alcea rosea* L. However, in horchata infusion, among the 23 compounds, containing malva esencia (*Malva* sp.), malva blanca (*Althea officinalis* L.), and pena pena (*Fuchsia laxensis* Kunth), thirteen quercetin glycoside were detected, like quercetin-O-pentoside, querecetin-O-galactoside, or methylquerce-tin-O-pentosyl-hexoside-O-hexoside [64]. Other authors have observed the abundance of quercetin and kaempferol glycosides in *C. oleifera* and *C. polyodonta* flowers [69].

In horchata infusion, two flavonones, hesperetin-O-caffeoyl-deoxyhexosyl-hexoside and naringenin hexoside, and two anthocyanins, Cyanidin-O-coumaroylhexosyl-O-hexoside and Cyanidin-O-coumaroylhexosyl-O-hexoside [68] were also detected.

As other edible flowers [70], pena pena and malvagoma can be considered a source of anthocyanins. Their values, especially those shown in pena pena flower powder, are higher than those shown in the main considered sources of anthocyanins, like berries, purple corn, cherries, plums, eggplant, wine, grapes, black carrots, red cabbage, and purple cauliflower, whose contents ranged between 1.0 and 14 mg/g [71]. Worldwide, the compound annual growth rate of anthocyanin food colorants is projected to be 4.8% from 2022 to 2032, owing to the widespread acceptance of natural pigments over synthetic ones [17,72,73]. In this sense, edible flowers have become a valuable resource for extracting pigments such as anthocyanins, betalains, carotenoids, and many other non-anthocyanin compounds that can be used in food formulation. The obtention of stable powder of flowers could contribute to the inclusion of them in functional foods or as a source of food colorants or in the intelligent package.

## 4. Conclusions

The petals of *Alcea rosea* and *Fuchsia hybrida* contain high levels of carbohydrates and proteins. Additionally, noteworthy is the content of dietary fiber, which grants better hydration properties to malvagoma due to the significantly higher levels of this nutrient.

The total unsaturated fatty acids exceeded the total saturated fatty acids in these flowers. Linoleic acid (C18:2; n 6,9) and linolenic acid (C18:3; n 3,6,9) were the major fatty acids found, followed by palmitic acid (C16:0). Both flowers are sources of polyphenols, especially anthocyanins. Pena pena flower powder can be considered a source of ellagic acids and derivatives, while malvagoma highlights the content of flavones and luteolin glycoside.

The results obtained demonstrate the potential of these two flowers to be used in food formulation, potentially enhancing nutritional, sensory, and functional characteristics. Future research is necessary to delve deeper into the influence of transformation processes on functional components. Additionally, studies on digestibility are needed to understand the actual bioavailability of these components.

## Figures and Tables

**Table 1:** Composition of flower powders (g 100 g^−1^).

Components	Pena Pena (*Fuchsia hybrida*)	Malvagoma (*Alcea rosea* L.)
Moisture	11.47 ± 0.03 ^a^	8.67 ± 0.17 ^b^
Fat	1.76 ± 0.16 ^b^	2.67 ± 0.11 ^a^
Proteins	9.38 ± 0.02 ^b^	12.10 ± 0.05 ^a^
Ash	6.00 ± 0.02 ^b^	7.62 ± 0.02 ^a^
Total carbohydrates	71.55 ± 0.25 ^a^	68.95 ± 0.08 ^b^
Total dietary fiber	32.59 ± 1.00 ^b^	40.70 ± 1.21 ^a^
Glucose	5.16 ± 0.42 ^a^	4.13 ± 0.16 ^b^
Fructose	11.81 ± 0.97 ^a^	7.96 ± 0.46 ^b^

Results are expressed as a mean of three replicates ± standard deviation. Values with the same letter in the same row are not statistically different (*p* > 0.05).

**Table 2:** Physicochemical and techno-functional properties of flower powders.

Properties	Pena Pena (*Fuchsia hybrida*)	Malvagoma (*Alcea rosea* L.)
Water activity	0.448 ± 0.001 ^a^	0.390 ± 0.009 ^b^
pH	4.71 ± 0.03 ^b^	6.00 ± 0.01 ^a^
Density (Kg/m^3^)	287.2 ± 2.0 ^b^	355.3 ± 6.1 ^a^
OHC (g/g)	2.38 ± 0.04 ^a^	2.55 ± 0.12 ^a^
SWC (mL/g)	4.73 ± 1.72 ^b^	14.05 ± 1.01 ^a^
Color coordinates		
L*	53.39 ± 0.22 ^b^	55.49 ± 0.71 ^a^
a*	10.56 ± 0.15 ^a^	2.39 ± 0.07 ^b^
b*	7.64 ± 0.10 ^a^	6.79 ± 0.32 ^b^
C*	13.04 ± 0.07 ^a^	7.20 ± 0.30 ^b^
h°	35.89 ± 0.74 ^b^	70.57 ± 1.05 ^a^

Results are expressed as a mean of three replicates ± standard deviation. OHC—oil holding capacity, SWC—swelling capacity, L*—lightness, a*—green-red components, b*—blue d—yellow components, C*—chroma, h°—hue. Values with the same letter in the same row are not statistically different (*p* > 0.05).

**Table 3:** Fatty acid profile of flower powders (mg/100 g dw).

Fatty Acids		Pena Pena (*Fuchsia hybrida*)	Malvagoma (*Alcea rosea* L.)
SFA			
C6:0	Caproic acid	0.83 ± 0.01 ^a^	0.53 ± 0.05 ^b^
C8:0	Caprylic acid	1.85 ± 0.54 ^a^	0.82 ± 0.03 ^a^
C10:0	Capric acid	2.74 ± 0.41 ^a^	0.82 ± 0.04 ^b^
C12:0	Lauric acid	1.88 ± 0.27 ^b^	4.41 ± 0.50 ^a^
C13:0	Isomorphic acid	1.40 ± 0.13 ^b^	4.79 ± 0.50 ^a^
C14:0	Myristic acid	13.17 ± 0.49 ^b^	19.69 ± 1.01 ^a^
C15:0	Pentadecanoic acid	2.88 ± 0.13 ^a^	2.93 ± 0.05 ^a^
C16:0	Palmitic acid	267.31 ± 5.27 ^b^	392.49 ± 4.66 ^a^
C17:0	Margigaric acid	2.90 ± 0.17 ^b^	5.14 ± 0.12 ^a^
C18:0	Stearic acid	44.97 ± 0.20 ^a^	99.92 ± 14.56 ^a^
C20:0	Arachidic acid	42.56 ± 1.50 ^b^	86.83 ± 0.49 ^a^
C22:0	Docosanoic acid	17.29 ± 0.67 ^b^	41.53 ± 0.10 ^a^
C23:0	Tricosanoic acid	nd ^b^	9.03 ± 0.54 ^a^
C24:0	Tetracosanoic acid	9.77 ± 0.33 ^b^	24.62 ± 0.17 ^a^
MUFA			
C14:1	Myristoleic acid	nd ^b^	8.75 ± 1.08 ^a^
C16:1	Palmitoleic acid	3.25 ± 0.18 ^b^	4.51 ± 0.19 ^a^
C17:1	cis-10-heptadecenic acid	nd ^b^	19.06 ± 0.16 ^a^
C18:1	Oleic acid	70.14 ± 1.70 ^b^	181.96 ± 1.15 ^a^
C20:1	Gadoleic acid	8.93 ± 2.65 ^a^	5.30 ± 0.15 ^b^
PUFA			
C18:2 (n 6,9)	Linoleic acid	599.97 ± 14.20 ^a^	286.38 ± 1.02 ^b^
C18:2 (n 3,6)	Linolelaidic acid	5.61 ± 0.47 ^b^	46.70 ± 3.10 ^a^
C18:3 (n 3,6,9)	Linolenic acid	280.43 ± 10.92 ^b^	496.65 ± 6.64 ^a^
C18:3 (n 6,9,12)	Gamma- linolenic acid	nd ^b^	38.55 ± 1.95 ^a^
C20:2 (n 11,14)	Eicosadienoic acid	4.16 ± 0.27 ^b^	104.35 ± 8.42 ^a^
C20:4 6,9,12,15	Arachidonic acid	nd ^b^	61.55 ± 8.06 ^a^
∑SFA		409.55 ± 6.31 ^b^	693.54 ± 8.46 ^a^
∑MUFA		82.32 ± 0.77 ^b^	253.48 ± 0.28 ^a^
∑PUFA		894.58 ± 25.89 ^b^	1043.32 ± 7.51 ^a^
∑n-3		286.04 ± 11.4 ^b^	543.36 ± 9.75 ^a^
∑n-6		599.97 ± 14.2 ^a^	386.48 ± 8.99 ^b^

Results are expressed as a mean of three replicates ± standard deviation. nd—not detected. SFA—saturated fatty acids, MUFA—monounsaturated fatty acids, PUFA—polyunsaturated fatty acids. Values with the same letter in the same row are not statistically different (*p* > 0.05).

**Table 4:** (Poly)phenol profile of pena pena (*Fuchsia hybrida*) flower powder (µg/g).

		Free	Bound
Hydroxybenzoic acids and derivatives	Ellagic acid	3601.1 ± 728.1	458.1 ± 31.39
	Ellagic acid glycoside I		12.5 ± 2.52
	Ellagic acid glycoside II		4.9 ± 0.17
	Ellagic acid glycoside III		11.5 ± 0.18
	Ellagic acid glycoside IV		16.0 ± 3.22
	Ellagic acid glycoside V		24.0 ± 6.16
	Ellagic acid glycoside VI		260.8 ± 34.73
	Gallic acid	1383.2 ± 208.0	4075.1 ± 244.86
	Syringic acid	650.9 ± 176.3	
Hydroxycinnamic acids and derivatives	Caffeic acid		16.5 ± 1.53
	Ferulic acid		17.3 ± 2.08
	*p*-Coumaric acid		14.0 ± 2.60
	*p*-Coumaric acid derivative		4.4 ± 0.77
Anthocyanins	Cy-3-O-β-glucopyranoside	115.6 ± 5.5	
	Dp 3-O-β-glucopyranoside	81.8 ± 7.3	
	Mv 3-O-β-glucopyranoside	188.8 ± 26.5	
	Pg 3-O-β-glucopyranoside	8311.5 ± 1472.9	
	Pn 3-O-β-glucopyranoside	72.2 ± 6.6	
	Pt 3-O-β-glucopyranoside	129.8 ± 13.3	
Flavone	Apigenin glycoside I	3.5 ± 0.2	
	Luteolin glycoside I	337.2 ± 63.0	465.9 ± 33.32
	Luteolin glycoside II	218.4 ± 2.8	412.7 ± 26.80
	Luteolin glycoside III	56.4 ± 4.4	
	Luteolin glycoside IV	51.6 ± 11.3	
	Luteolin glycoside V	153.7 ± 27.6	127.4 ± 8.99
Flavonols	Kaempherol		4.3 ± 0.11
	Kaempherol glycoside I		13.6 ± 1.15
	Quercetin	1.6 ± 0.0	4.8 ± 0.37
	Quercetin glycoside I	774.5 ± 166.3	
	Quercetin glycoside II	851.0 ± 170.2	
	Quercetin glycoside III	3717.4 ± 405.7	
	Quercetin glycoside V	5.3 ± 1.1	
	Quercetin glycoside VI	190.7 ± 38.3	87.1 ± 11.42
	Quercetin glycoside VII	39.9 ± 6.9	
	Rutin	1525.0 ± 102.3	

Results are expressed as a mean of three replicates ± standard deviation. Cy: Cyanidin; Dp: Delphinidin Mv: Malvidin; Pg: Pelargonidin; Pt: Petunidin; Pn peonidin.

**Table 5:** (Poly)phenol profile of malvagoma (*Alcea rosea* L.) flower powder (µg/g).

		Free	Bound
Hydroxybenzoic acids and derivatives	4-Hydroxybenzoic acid		14.8 ± 2.1
	Gallic acid		8.1 ± 0.0
	Protocatechuic acid		4.1 ± 0.4
	Protocatechuic derivative	33.1 ± 4.3	
	Syringic acid		20.4 ± 3.7
Hydroxycinnamic acids derive	Caffeic acid		467.9 ± 49.9
	Caffeic derivative I	18.7 ± 0.5	
	Caffeic derivative II	13.9 ± 4.4	
	Chlorogenic acid	199.8 ± 18.4	
	Chlorogenic acid derivative	49.4 ± 1.4	
	Ferulic acid		359.3 ± 38.0
	Ferulic derivative		9.1 ± 0.3
	*p*-Coumaric acid		234.0 ± 29.2
	Hydrocinnamic acid	174.2 ± 152.4	
Anthocyanins	Cy-3-O-β-glucopyranoside	147.2 ± 17.4	
	Mv-3-O-β-glucopyranoside	710.9 ± 66.6	
	Mv-3,5-O- β-glucopiyranoside	590.3 ± 62.4	
	Pg-3-O-β-glucopyranoside	570.0 ± 79.0	
	Pt-3-O-β-glucopyranoside	995.1 ± 105.2	
	Anthocyanin I	1179.0 ± 152.4	
	Anthocyanin II	348.5 ± 51.6	
	Anthocyanin III	868.3 ± 80.8	
Flavanols	Gallocatechin gallate		534.9 ± 54.3
Flavanone	Naringin glycoside I	3597.6 ± 659.0	84.8 ± 36.9
	Naringin glycoside II	12,078.7 ± 1880.3	308.4 ± 134.3
Flavone	Apigenin glycoside II	138.4 ± 33.9	
	Apigenin glycoside III	32.7 ± 3.0	
	Luteolin glycoside II	7224.1 ± 914.4	1758.3 ± 548.3
Flavonols	Kaempherol	41.7 ± 2.3	
	Kaempherol glycoside II	132.6 ± 14.1	12.2 ± 3.4
	Kaempherol glycoside III	64.7 ± 9.0	6.9 ± 0.8
	Quercetin glycoside III	570.5 ± 81.4	21.6 ± 9.1
	Quercetin glycoside IV	236.3 ± 34.7	
	Quercetin glycoside VIII	42.8 ± 7.7	
	Quercetin methyl-glycosyde I	236.5 ± 57.8	
	Quercetin methyl-glycosyde II	173.3 ± 29.6	
	Rutin	619.8 ± 94.1	

Results are expressed as a mean of three replicates ± standard deviation. Cy: Cyanidin; Mv: Malvidin; Pg: Pelargonidin; Pt: Petunidin.

## Data Availability

Data are contained within the article.

## Supplementary Materials

The following supporting information can be downloaded at: https://www.mdpi.com/article/10.3390/foods13020237/s1.
