# Supplementary material for: Nutritional, Fatty Acids, (Poly)phenols and Technological Properties of Flower Powders from Fuchsia hybrida and Alcea rosea
Source: Foods. 2024 Jan 11;13(2):237. doi: 10.3390/foods13020237 (PMC10814466; doi:10.3390/foods13020237)
Supplement: Supplementary file 1 [file foods-13-00237-s001.zip › foods-2789640-supplementary.pdf]

Nutritional, Fatty Acids, (Poly)phenols and Technological Properties of Flower Powders from *Fuchsia hybrida* and *Alcea rosea*

Maritza Castillo-Carrión[1], Ruth Martínez-Espinosa[1], José Ángel Pérez-Ángel[2], Juana Fernández-López[2], Manuel Viuda-Martos[2], Raquel Lucas-González[2,3]*

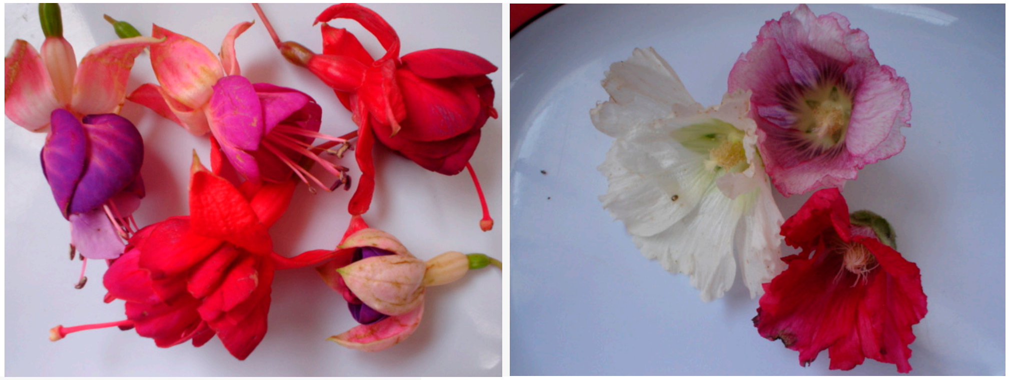

(a) (b)

Supplementary Figure S1. Edible flowers studied: (a) Pena pena (*Fuchsia hybrida*), (b) Malvagoma (*Alcea rosea L*)

Supplementary Table S1. Detected (poly)phenols free and bound fractions Pena pena (*Fuchsia hybrida*) and Malvagoma (*Alcea rosea L*) flower powders.

**Nº**	**rt (min)**	**Tentative compound**	**UV bands (nm)**	**Calibration curve used for quantification**	Fr.	**Pena pena** (*Fuchsia hybrida*)	Fr.	**Malvagoma** (*Alcea rosea L.)*
1	7.4	Gallic acid*	234 270	Gallic acid	F/B	x	B	x
2	8.2	Caffeic acid derivative I	242 sh 290 324	Caffeic acid	-	-	F	x
3	8.6	Protocatechuic derivative	232 260 292	Protocatechuic acid	-	-	F	x
4	10.7	Gallocatechin gallate*	236 270	Gallocatechin gallate	-	-	B	x
5	11.3	Chlorogenic acid derivative	244 sh 298 328	Chlorogenic acid	-	-	F	x
6	11.6	Protocatechuic acid*	232 260 292	Protocatechuic acid	-	-	B	x
7	12.1	Ellagic acid glycoside I	254 368	Ellagic acid	B	x	-	-
9	13.9	Pelargonidin 3-O-β-glucopyranoside*	238 278 sh452 522	Pelargonidin 3-O-β-glucopyranoside	F	x	F	x
10	14.2	Malvidin-3,5-O-B-glucopiyranoside*	276 526	Malvidin-3,5-O-B-glucopiyranoside	-	-	F	x
11	15.4	Cyanydin-3-O-β-glucopyranoside*	238/280/sh452 520	Cyanydin-3-O-β-glucopyranoside	F	x	F	x
12	15.5	Chlorogenic acid*	244 sh 298 326	Chlorogenic acid	-	-	F	x
14	15.9	Peonidin 3-O-β-glucopyranoside*	276 526	Peonidin 3-O-β-glucopyranoside	F	x	-	-
15	15.9	4-Hydroxybenzoic acid*	228 256	4-Hydroxybenzoic acid	-	-	B	x
16	16.1	Anthocyanin I	236 276 526	Malvidin-3,5-O-B-glucopiyranoside	-	-	F	x
17	16.7	Delphinidin 3-O-β-glucopyranoside*	276 502	Delphinidin 3-O-β-glucopyranoside	F	x	-	-
18	16.7	Ellagic acid glycoside II	254 368	Ellagic acid	B	x	-	-
19	16.8	Hydrocinnamic acid	244 sh290 330	phenolic acid	-	-	F	x
20	17.0	Ellagic acid glycoside III	254 368	Ellagic acid glycoside	B	x	-	-
21	17.5	Petunidin 3-O-β-glucopyranoside*	240 280 sh250 518	Petunidin 3-O-β-glucopyranoside	F	x	F	x
22	17.8	Malvidin 3-O-β-glucopyranoside*	244 276 sh260 530	Malvidin 3-O-β-glucopyranoside	F	x	F	x
23	17.8	Caffeic acid*	242 sh 290 324	Caffeic acid	B	x	B	x

24	17.9	Ellagic acid glycoside IV	254 368	Ellagic acid glycoside	B	x	-	-
25	18.3	Quercetin methyl-glycosyde I	256 370	Quercetin methyl-glycosyde	-	-	F	x
26	18.2	Ellagic acid glycoside V	254 368	Ellagic acid glycoside	B	x	-	-
27	18.3	Syringic acid*	230 274	Syringic acid*	F	x	B	x
28	18.7	Ellagic acid glycoside VI	254 368	Ellagic acid glycoside	B	x	-	-
29	19.0	Quercetin methyl-glycosyde II	256 370	Quercetin methyl-glycosyde	-	-	F	x
30	19.9	Naringin glycoside I	236 290	Naringin derive	-	-	F/B	x
32	20.8	Naringin glycoside II	236 290	Naringin derive	-	-	F/B	x
33	20.9	Quercetin glycoside I	256 356	Quercetin	F	x	-	-
35	22.4	Rutin*	256 356	Rutin	F	x	F	x
36	22.7	*p*-Coumaric acid*	sh300 310	p-Coumaric acid	B	x	B	x
37	23	Quercetin glycoside II	256 356	Quercetin	F	x	-	-
38	23.8	Quercetin glycoside III	256 356	Quercetin	F	x	F/B	x
39	23.6	Ellagic acid*	254 sh304 368	Ellagic acid	F/B	x	-	-
40	24.2	Ferulic acid*	242 sh294 324	Ferulic acid	B	x	B	x
41	24.8	Apigenin glycoside I	268 338	Apigenin	F	x	-	-
42	24.9	Anthocyanins II	236 270 530	Malvidin	-	-	F	x
43	25.3	Luteolin glycoside I	252 266 348	Luteolin	F/B	x	-	-
44	25.2	Quercetin glycoside IV	256 356	Quercetin	-	-	F	x
45	25.7	Quercetin glycoside V	256 356	Quercetin	F	x	-	-
46	25.8	p-Coumaric acid derivative	300 310	p-Coumaric acid	B	x	-	-
47	26	Luteolin glycoside II	252 266 348	Luteolin	F/B	x	F/B	x
48	26.3	Quercetin glycoside VI	256 356	Quercetin	F/B	x	-	-
49	26.4	Anthocyanins III	236 270 530	Malvidin	-	-	F	x
50	26.6	Quercetin glycosideVII	256 356	Quercetin	F	x	-	-
51	26.6	Kaempherol glycoside I	266 366	Kaempherol	B	x	-	-
52	26.9	Apigenin glycoside II	268 336	Apigenin	-	-	F	x
53	27.1	Luteolin glycoside III	252 266 348	Luteolin	F	x	-	-

54	27.4	Kaempherol glycoside II	266 366	Kaempherol	-	-	F/B	x
55	27.8	Quercetin glycoside VIII	256 356	Quercetin	-	-	F	x
56	28	Luteolin glycoside IV	252 266 348	Luteolin	F	x	-	-
57	28.2	Ferulic derivative	242 sh294 324	Ferulic	-	-	B	x
58	28.8	Luteolin glycoside V	252 266 250	Luteolin	F/B	x	-	-
59	29.5	Kaempherol glycoside III	266 366	Kaempherol	-	-	F/B	x
60	31.6	Caffeic acid derivative II	242 sh 290 324	Caffeic acid	-	-	F	x
61	33.6	Quercetin*	256 370	Quercetin	F/B	x	-	-
62	33.9	Apigenin glycoside III	268 338	Apigenin	-	-	F	x
63	38.4	Kaempherol*	250 266 366	Kaempherol	B	x	F	x
				**Total bound**	12		9	
				**Total free**	18		22	
				**Total both**	7		6	
				**Total quantified compounds**	37		37	

*Compounds confirmed by standard
